# HIF prolyl hydroxylase PHD3 regulates translational machinery and glucose metabolism in clear cell renal cell carcinoma

**DOI:** 10.1186/s40170-017-0167-y

**Published:** 2017-07-04

**Authors:** Petra Miikkulainen, Heidi Högel, Krista Rantanen, Tomi Suomi, Petri Kouvonen, Laura L. Elo, Panu M. Jaakkola

**Affiliations:** 1 Turku Centre for Biotechnology, University of Turku and Åbo Akademi University, Tykistökatu 6, 20520 Turku, Finland; 2 Department of Medical Biochemistry, Faculty of Medicine, University of Turku, Kiinamyllynkatu 10, 20520 Turku, Finland; 3 Department of Information Technology, Faculty of Mathematics and Natural Sciences, University of Turku, Vesilinnantie 5, 20520 Turku, Finland; 4 Department of Oncology and Radiotherapy, Turku University Hospital, Hämeentie 11, 20520 Turku, Finland

**Keywords:** ccRCC, PHD3, Discovery proteomics, Hypoxia, Metabolism, Ribosomal proteins, Translation

## Abstract

**Background:**

A key feature of clear cell renal cell carcinoma (ccRCC) is the inactivation of the von Hippel-Lindau tumour suppressor protein (pVHL) that leads to the activation of hypoxia-inducible factor (HIF) pathway also in well-oxygenated conditions. Important regulator of HIF-α, prolyl hydroxylase PHD3, is expressed in high amounts in ccRCC. Although several functions and downstream targets for PHD3 in cancer have been suggested, the role of elevated PHD3 expression in ccRCC is not clear.

**Methods:**

To gain insight into the functions of high PHD3 expression in ccRCC, we used PHD3 knockdown by siRNA in 786-O cells under normoxic and hypoxic conditions and performed discovery mass spectrometry (LC-MS/MS) of the purified peptide samples. The LC-MS/MS results were analysed by label-free quantification of proteome data using a peptide-level expression-change averaging procedure and subsequent gene ontology enrichment analysis.

**Results:**

Our data reveals an intriguingly widespread effect of PHD3 knockdown with 91 significantly regulated proteins. Under hypoxia, the response to PHD3 silencing was wider than under normoxia illustrated by both the number of regulated proteins and by the range of protein expression levels. The main cellular functions regulated by PHD3 expression were glucose metabolism, protein translation and messenger RNA (mRNA) processing. PHD3 silencing led to downregulation of most glycolytic enzymes from glucose transport to lactate production supported by the reduction in extracellular acidification and lactate production and increase in cellular oxygen consumption rate. Moreover, upregulation of mRNA processing-related proteins and alteration in a number of ribosomal proteins was seen as a response to PHD3 silencing. Further studies on upstream effectors of the translational machinery revealed a possible role for PHD3 in regulation of mTOR pathway signalling.

**Conclusions:**

Our findings suggest crucial involvement of PHD3 in the maintenance of key cellular functions including glycolysis and protein synthesis in ccRCC.

**Electronic supplementary material:**

The online version of this article (doi:10.1186/s40170-017-0167-y) contains supplementary material, which is available to authorized users.

## Background

Clear cell renal cell carcinoma (ccRCC), the most common subtype of human kidney carcinomas, is commonly associated with the inactivating mutations of *VHL* leading to a loss of function of the von Hippel‐Lindau tumour suppressor protein (pVHL). This leads to constant activation of hypoxia signalling pathway paving the way for malignant progression (reviewed in [1–3]). Among other malignant characteristics, a pronounced Warburg effect—cancer cells utilizing aerobic glycolysis as primary pathway of energy production—is a common feature of ccRCC tumours and derived cell lines [4–6]. As shown by the expression level data and metabolomics profiling, in ccRCC, the metabolic switch to increased glycolysis and lactate production correlates with cancer aggressiveness and poor patient prognosis [6, 7].

Hypoxia-inducible factors (HIFs), consisting of HIF-α and HIF-β/ARNT subunits, are the main transcription factors mediating the adaptive responses to hypoxia by activating transcription of their target genes (reviewed in [8]). HIF-α subunits are regulated by a family of prolyl-4-hydroxylases (PHD1-3) that hydroxylate key proline residues in HIF-α in an oxygen-dependent manner. Thus, HIF-α is marked for pVHL-dependent ubiquitination and proteasomal degradation under sufficient oxygen availability. However, in the presence of pVHL loss of function, the HIF-α subunits are not degraded, leaving them constantly expressed. This constitutive expression of HIF-α leads to the activation of several hundred HIF target genes acting on angiogenesis, proliferation, survival, metabolism and apoptosis pathways. The activation of hypoxia signalling in ccRCC is known to be strongly oncogenic and as such an essential driver for tumour growth (reviewed in [2, 3]). HIF-1α and HIF-2α subunits have shown to activate distinct but overlapping set of target genes. Their activation is dependent on cell type [9, 10]. As compared to other cancer types, in ccRCC, the differential activation of target genes leads to contrasting effects on cancer progression, as high HIF-2α expression enhances tumorigenic activity, whereas high HIF-1α has tumour suppressive effect [11–13].

Among the three PHD family members, PHD3 (also known as EGLN3) shows the most robust hypoxic induction and it has been suggested to partly retain the enzymatic activity under hypoxia (reviewed in [14]). Besides HIF-α, PHD3 has also been suggested to have a variety of hydroxylation targets as well as hydroxylation-independent functions. The reported functions of PHD3 include the regulation of cellular survival mechanisms via regulation of apoptosis, cell cycle and NF-κB signalling [15–18]. We have previously reported that PHD3 is needed for the hypoxic cell cycle to proceed over G1/S checkpoint [18] by reducing the stability of cyclin-dependent kinase inhibitor p27 in human carcinoma cells including ccRCC cells [19]. PHD3 has also been linked to cancer cell metabolism by regulation of pyruvate kinase M2 (PKM2) in two distinct mechanisms [20, 21]. Most recently, PHD3 has been suggested to function as an activator of fatty acid oxidation via ACC2 [22]. PHD3 overexpression has been seen in many types of cancer, including ccRCC [23, 24]. Importantly, high PHD3 expression serves as a marker for poor prognosis in ccRCC patients [25] as well as in other types of cancer [26].

Although several functions and downstream targets for PHD3 in cancer have been suggested, the role of elevated PHD3 expression in ccRCC is far from clear. PHD3 can mediate its HIF-dependent functions to the gene expression level, but the mechanism of HIF-independent functions of PHD3 is less clear. As the protein-level data represents the functional level in cellular processes and signal transduction, we selected a discovery proteomics approach to gain an overall insight into the functions of high PHD3 expression in ccRCC cell line. Discovery proteomics is a widely used method consisting of a liquid chromatography (LC) system connected to a tandem mass spectrometer operated in electrospray ionizing mode (LC-MS/MS). In LC-MS/MS, the peptide components of the sample are separated by liquid chromatography and analysed by mass spectrometer followed by a database search to convert peptide information into a proteome-level data. Furthermore, we exploited the possibility of label-free quantification of proteome-level data, with a novel method of peptide-level expression-change averaging procedure [27].

Here, we show that PHD3 depletion significantly affects the essential cellular processes related to glucose metabolism, post-transcriptional modification and ribosomal subunits and translation regulation in ccRCC cells. This study provides novel insights into the role of PHD3 in ccRCC by revealing functional groups of proteins that respond to PHD3 knockdown.

## Methods

### Cell culture and transfections

786-O cells were obtained from ATCC (Rockville, MD, USA) (purchased 10/2013) and cultured in RPMI-1640 medium (Lonza). RCC4 (*VHL* ^*−/−*^ *)* cells were acquired from Prof. Peppi Karppinen (University of Oulu, Finland) and cultured in Dulbecco’s modified Eagle’s medium (DMEM, Sigma-Aldrich). Both media were supplied with 10% fetal bovine serum (FBS, Biowest), l-glutamine (Lonza) and penicillin/streptomycin (Lonza). Cells were cultured in a humidified atmosphere in 37 °C containing 5% CO_2_ and for hypoxic experiments in 1% O_2_ in a hypoxic workstation (Invivo_2_, Ruskinn Technology) with oxygen replaced by 99.5% pure N_2_ (AGA, Finland). Cells were tested monthly for mycoplasma contamination when growing. For short interfering RNA (siRNA) transfections, two stranded oligonucleotides were used at final concentration of 10 nM. Reverse transfections were performed using Lipofectamine® RNAiMAX (Invitrogen) according to manufacturer’s protocol. The siRNAs (MWG Biotech AG) used were the following: non-target (Scr) 5′-CCUACAUCCCGAUCGAUGAUG(dTdT)-3′, siPHD3#1 5′-GUCUAAGGCAAUGGUGGC-UUG(dTdT)-3′ and siPHD3#2 5′-AGGAGAGGUCUAAGGCAAUG(dTdT)-3′. For rapamycin treatment, transfected cells were cultured in normoxic or hypoxic condition for 20 h followed by 4 h of rapamycin (100 nM) in fresh cell culture media.

### Western blot analysis

Cells were harvested in SDS-Triton lysis buffer with protease inhibitors. Protein concentration was measured using Bio-Rad DC Protein assay. Equal amounts of protein were loaded and run on SDS-PAGE and transferred to a PVDF membrane (Millipore). Western blot analyses with the following antibodies were performed: PHD3 (NB100-139, Novus Biologicals), CD70 (CD27 Ligand) (MAB2738, R&D Systems), Integrin β1 (610468, BD Transduction Laboratories), LDHA (#3582, Cell Signaling Technologies), MDH2 (ab181873, Abcam), STAT1 (#9176, Cell Signaling Technologies), Fibronectin (F3648, Sigma), alpha-actinin 4 (ALX-210-356-C050, Enzo Life Sciences), GLUT1 (ab14683, Abcam), glyceraldehyde phosphate dehydrogenase (GAPDH) (5G4-6C5, HyTest), S6 ribosomal protein (#2217, Cell Signaling Technologies), pS6 ribosomal protein S235/236 (#2211, Cell Signaling Technologies), pS6 ribosomal protein S240/244 (#3564, Cell Signaling Technologies), p70 S6 kinase (#2708, Cell Signaling Technologies), p-p70 S6 kinase T389 (#9234, Cell Signaling Technologies), α-tubulin (sc-23948, Santa Cruz Biotechnology), β-actin (AC-74, Sigma-Aldrich), anti-mouse-HRP (DAKO), and anti-rabbit HRP (DAKO). Protein detection was performed using Pierce ECL Western blotting substrate (Thermo Fisher Scientific). β-actin, α-tubulin and GAPDH were used as loading controls.

### RT-PCR

For real-time PCR, RNA was extracted using NucleoSpin RNA II kit (Macherey-Nagel) and reverse transcription was performed using M-MuLV RNase H-reverse transcriptase (Finnzymes, Thermo Fisher Scientific) according to the manufacturer’s protocol. RT-PCR reactions were run using QuantStudio 12K Flex (Thermo Fisher Scientific) and TaqMan Universal Master Mix II, no UNG (Applied Biosystems, Life Technologies). The TaqMan primers (Oligomer) used for PHD3 were the following: atcgacaggctggtcctcta (left), gatagcaagccaccattgc (right) and probe (Roche, Universal ProbeLibrary) #61 (cat. no. 04688597001). PHD3 mRNA expression was normalized against the expression of glyceraldehyde phosphate dehydrogenase (*GAPDH*).

### Gel electrophoresis and in-gel digestion

Proteins were separated on Criterion XT 12% Bis-Tris gel (Bio-Rad) and silver stained. Bands were cut into 1-mm pieces and reduced and alkylated with 20 mM dithiothreitol (DTT) in 100 mM NH_4_HCO_3_ and with 55 mM iodoacetamide (IAA) in 100 mM NH_4_HCO_3_, respectively. For reaching optimal efficacy of the reduction and alkylation, the samples were incubated in DTT for 30 min in +56 °C and in IAA for 20 min in RT, dark. In between the reagents, gel pieces were dehydrated with 100% acetonitrile (ACN). After reduction and alkylation, 60 μl of trypsin in 40 mM NH_4_HCO_3_/10% ACN was added and incubated in +37 °C for 18 h.

After in-gel digestion, the peptides were extracted in two steps: first, using 100% ACN for 15 min in +37 °C and, second, using 50% ACN in 5% CHOOH for 15 min in +37 °C followed by a mixing of the supernatants. The extracted peptides were vacuum dried and stored at −20 °C until analysis.

### LC-MS/MS analysis

Tryptic peptides were dissolved in 0.2% formic acid (CHOOH), and 200-ng samples were submitted for LC-MS/MS system, where peptides were separated according to their hydrophobicity on a reversed-phase chromatography column. Each sample was analysed in three biological replicates using a QExactive hybrid quadrupole-Orbitrap mass spectrometer (Thermo Fisher Scientific). The QExactive was coupled to an EASY-nLC 1000 nano flow LC instrument (Thermo Fisher Scientific). Sample loading, solvent delivery and scan functions were controlled by Xcalibur software (version 2.1.0 SP1.1160, Thermo Fisher Scientific). An in-house-build trap column (2.5 cm long, 75 μm inner diameter) (Magic AQ C18 resin, 5 μm/200 Å, Bruker-Michrom) was used for desalting and concentrating the sample, and an analytical column (15 cm long, 75 μm inner diameter) (PicoFrit, 15 μm, NewObjective) with the same C18 resin was used for peptide separation. A 45-min gradient from 95% solvent A (98% H_2_O, 2% ACN and 0.2% HCOOH) to 90% solvent B (95% ACN, 5% H_2_O and 0.2% HCOOH) with a flow rate 0.3 μL/min was used for peptide elution.

Data-dependent acquisition was performed in positive ion mode. The MS spectra were acquired from the range of 300–2000 *m*/*z* in the Orbitrap with resolution of 70,000, an AGC target value of 1 × 10^6^ ions and a maximal injection time of 120 ms. The MS/MS spectra were acquired in the Orbitrap with a resolution of 17,500, isolation window of 2.0 *m*/*z*, an AGC target value of 2 × 10^4^ ions, maximal injection time of 250 ms, lowest mass fixed at 100 *m*/*z* and dynamic exclusion duration set to 15 s.

The database search for the raw spectrum files was performed in Proteome Discoverer (version 1.3.0.339, Thermo Fisher Scientific) by using Mascot (Matrix Science). The search was done for peptides formed with trypsin digestion, where one missed cleavage site was allowed, against UniProtKB/Swiss-Prot human database (2015-05-26). Search parameters were as follows: decoy database search was performed, accepted precursor mass tolerance was set to 5 ppm, and fragment mass tolerance to 0.02 Da, fixed modification of carbamidomethylation of cysteine and variable modification of methionine oxidation. The false discovery rate threshold was set to 1% by the Percolator algorithm.

### Data processing and analysis

Progenesis QI for proteomics (Nonlinear Dynamics) was used for quantification. The analysis area of the gradient was set to 0–45 min. Automatic peak picking was used in default sensitivity mode. Peptide abundances were exported from the software and median normalized. Differential expression between the sample groups was determined using the R-package PECA [27]. A paired test was performed using modified *t* test settings. PECA ranks the peptides of each protein by their *p* value and direction of change and uses the median *p* value of the ranked list as a score. Protein-level significance is then determined from the beta distribution using the score and the total number of peptides per each protein. Finally, the Benjamini-Hochberg procedure was used to calculate the false discovery rate (FDR).

### Pathway and functional group analysis

Gene ontology (GO) terms for genes coding for the significantly differentially expressed proteins were analysed for statistical over-representation (enrichment) of biological process using DAVID (Database for Annotation, Visualization and Integrated Discovery) 6.7 [28]. The analysed protein-coding genes were compared against a whole organism (Homo sapiens) background and with the highest classification stringency. Enrichment score was used to rank the annotation groups, and only groups with enrichment score above 1.3 (*p* value <0.05) were considered.

Known interactions between selected proteins were retrieved from the STRING interaction database [29]. Only high confidence (>0.7) interactions derived from experimental data or curated databases were considered.

### Functional assays on metabolic function

Transfected cells were cultured 24 h in normoxic (21% O_2_) condition followed by 24 h of normoxic or hypoxic (1% O_2_) condition, and the secreted lactate concentration was measured from cell culture medium by using l-Lactate colorimetric assay (Abcam). Cells were stained for nuclear stain Hoechst 33342, and lactate concentration was normalized to cell count.

Cell culture medium pH was measured from transfected cells cultured 24 h in normoxic condition followed by 24 h of normoxic or hypoxic condition. For pH measurements, the cells were stained for nuclear stain Hoechst 33342 and hydronium ion [H_3_O^+^] concentration was normalized to cell count and converted to pH.

Transfected cells were seeded (1 × 10^4^) on the 8-well Seahorse XFp Miniplate (Agilent Technologies) in triplicates. Prior analysis, cells were incubated for 1 h in XF Base medium (Agilent Technologies) supplemented with glucose, glutamine and pyruvate in a non-CO_2_ incubator at 37 °C. Seahorse XFp Analyser (Agilent Technologies) and Seahorse XF Cell Mito Stress Test Kit was used for determining the oxygen consumption rate (OCR) and glycolysis function by using the manufacturer’s protocol.

### 3D colony growth

Transfected cells were embedded in Matrigel® (Corning) on 96 wp, 1 × 10^4^ cells per well in triplicates. Culture medium was applied on top of the Matrigel® and changed in every 2 days. 786-O cells were grown for 7 days and RCC4 cells for 10 days after which the wells were imaged with a phase contrast microscope with ×2 objective. Average colony size was quantified from each well using ImageJ (NIH) from three (786-O) or two (RCC4) biological replicates with triplicate wells of each siRNA treatment. Images in the representative image panel was acquired by using ×4 objective, scale bar 100 μm.

### Statistical analysis

Quantified data from the WB, RT-PCR, functional assay metabolism, proliferation and 3D colony growth were reported as means together with their respective standard errors (SEM). Two-tailed paired *t* test was used to assess the statistical significance of differences between the siRNA and the corresponding control samples. Nominal *p* values were reported; single asterisk indicates *p* value <0.05, double asterisk indicates *p* value <0.01, triple asterisk indicates *p* value <0.001, and n.s. indicates not significant.

## Results

### Experimental setup and the effect of PHD3 silencing on 786-O global proteome

**Fig. 1:**
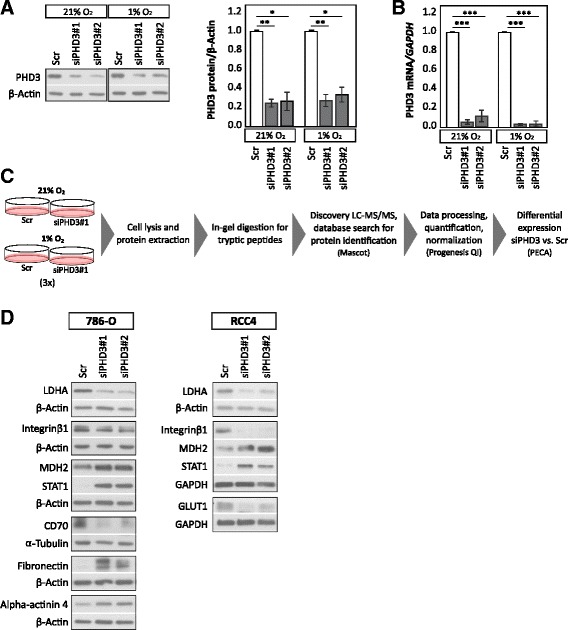
Experimental setup and the effect of PHD3 silencing on 786-O proteome. **a** siRNA-mediated silencing of PHD3 protein level in 786-O ccRCC cell line using two individual siRNA sequences in normoxic and in hypoxic (1% O_2_) condition. Quantification of three biological replicates, mean ± SEM, fold change to control (Scr). *Asterisk* indicates a statistically significant difference (*p < 0.05, **p < 0.01). **b** siRNA-mediated silencing of PHD3 mRNA expression, quantification of three individual experiments. Mean ± SEM, fold change to Scr (***p < 0.001). **c** Flow chart of the experimental procedure. 786-O cells were transfected with siPHD3#1 or with a non-targeting control siRNA (Scr) for 24 h followed by a hypoxic (1% O_2_) or normoxic (21% O_2_) exposure. Three independent experiments were performed; proteins were extracted, followed by in-gel digestion with trypsin. Purified peptides were ran through mass spectrometer. Protein identification was done with Mascot database search. Protein quantification was carried out with Progenesis QI, followed by testing the differential expression between sample groups using peptide-level expression change averaging (PECA). **d** Western blot validations of selected proteins in 786-O and RCC4 cell lines with two individual siRNA sequences targeting PHD3, representative analyses are shown

We undertook a task of characterizing the protein expression of ccRCC cell line 786-O using proteome-based techniques with PHD3 depletion. 786-O cell line has a high basal PHD3 expression both in normoxia and hypoxia. In addition, it expresses only the more oncogenic HIF-2α isoform but not HIF-1α. First, we tested two individual siRNA sequences for PHD3 silencing (siPHD3#1 and siPHD3#2), both well characterized by us and others [17–19, 30–32]. Both siRNAs knocked down both PHD3 protein and mRNA expression efficiently (siPHD3#1 and siPHD3#2 shown in Fig. 1a, b). siPHD3#1 was selected to be used for further experiments as it is the most widely used and the best verified sequence for its PHD3 specificity in the literature. Most importantly, the effect of both PHD3 siRNAs was verified for selected proteins acquired from the analysis of LC-MS/MS data (see Fig. 1d).

Figure 1c illustrates the used experimental procedure. 786-O cells were transfected with siPHD3#1 or with a non-targeting control siRNA (Scr) for 24 h followed by a hypoxic (1% O_2_) or normoxic exposure. Proteins were extracted, followed by in-gel digestion with trypsin. Purified peptides were injected into mass spectrometer through nano-flow liquid chromatographic system in three biological replicates as described in the “Methods” section. Subsequently, Mascot database search through Proteome Discoverer (Thermo Fischer Scientific) was used to identify proteins in each sample. Proteins identified with only one peptide were filtered out from further analysis. Protein quantification was carried out with Progenesis QI, followed by testing the differential expression between sample groups using peptide-level expression change averaging (PECA) [27], which performs statistical tests on peptide-level before estimating protein-level differential expression. Median normalization was applied to the data before performing modified *t* test and aggregating peptide-level *p* values into protein statistics.

For further studies, we chose only proteins that were differentially expressed between the conditions as estimated by PECA (false discovery rate FDR <0.05). The results of the PECA analysis are presented in Tables 1 and 2, for normoxic and hypoxic conditions, including UniProt accession numbers, protein and gene names, and values for log_2_ fold change and FDR. In total, we observed 63 significantly changed (FDR <0.05) proteins in hypoxic conditions and 28 proteins in normoxic conditions in response to PHD3 silencing. From these proteins, 45% were upregulated and 55% downregulated. Interestingly, under hypoxia, the range of the most upregulated and downregulated proteins appeared to be much larger (log_2_ fold change from 5.3 to −8.0) than under normoxia (log_2_ fold change from 1.5 to −1.6). Additional file 1: Figure S1 visualizes the peptide log_2_ fold changes of significantly changed proteins using box plots.

**Table 1:** Significantly regulated proteins in response to PHD3 silencing according to PECA analysis under normoxia

Accession	Protein name	Gene name	Log_2_ fold change	FDR
Upregulated				
P09493	Tropomyosin alpha-1 chain	TPM1 C15orf13 TMSA	1.5144	0.0260
P31943	Heterogeneous nuclear ribonucleoprotein H	HNRNPH1 HNRPH HNRPH1	1.3557	0.0020
P35527	Keratin, type I cytoskeletal 9	KRT9	1.0655	0.0124
Q15436	Protein transport protein Sec23A	SEC23A	0.9224	0.0092
P09429	High mobility group protein B1	HMGB1 HMG1	0.8762	0.0000
P51148	Ras-related protein Rab-5C	RAB5C RABL	0.7977	0.0016
O43852	Calumenin	CALU	0.6639	0.0007
P38646	Stress-70 protein, mitochondrial	HSPA9 GRP75 HSPA9B mt-HSP70	0.6142	0.0000
O75874	Isocitrate dehydrogenase	IDH1 PICD	0.5916	0.0116
O43707	Alpha-actinin-4	ACTN4	0.4702	0.0008
Q06210	Glutamine—fructose-6-phosphate aminotransferase	GFPT1 GFAT GFPT	0.4654	0.0449
P07195	l-lactate dehydrogenase B chain	LDHB	0.4116	0.0020
P55072	Transitional endoplasmic reticulum ATPase	VCP	0.3889	0.0002
P11413	Glucose-6-phosphate 1-dehydrogenase	G6PD	0.3781	0.0116
Downregulated				
P31939	Bifunctional purine biosynthesis protein PURH	ATIC PURH OK/SW-cl.86	−1.6058	0.0116
Q92597	Protein NDRG1	NDRG1 CAP43 DRG1 RTP	−1.3053	0.0000
O15460	Prolyl 4-hydroxylase subunit alpha-2	P4HA2 UNQ290/PRO330	−1.1904	0.0000
O43776	Asparagine—tRNA ligase, cytoplasmic	NARS	−0.9285	0.0000
Q6PIU2	Neutral cholesterol ester hydrolase 1	NCEH1 AADACL1 KIAA1363	−0.8525	0.0000
Q04637	Eukaryotic translation initiation factor 4 gamma 1	EIF4G1 EIF4F EIF4G EIF4GI	−0.8275	0.0275
P06733	Alpha-enolase	ENO1 ENO1L1 MBPB1 MPB1	−0.7775	0.0000
P11166	Glucose transporter 1	GLUT1 SLC2A1	−0.7694	0.0006
P54578	Ubiquitin carboxyl-terminal hydrolase 14	USP14 TGT	−0.7558	0.0114
P68363	Tubulin alpha-1B chain	TUBA1B	−0.7398	0.0016
P21980	Protein-glutamine gamma-glutamyltransferase 2	TGM2	−0.5013	0.0003
O00469	Procollagen-lysine,2-oxoglutarate 5-dioxygenase 2	PLOD2	−0.4866	0.0003
P35637	RNA-binding protein FUS	FUS TLS	−0.3575	0.0449
Q01082	Spectrin beta chain, non-erythrocytic 1	SPTBN1 SPTB2	−0.2911	0.0260

**Table 2:** Significantly regulated proteins in response to PHD3 silencing according to PECA analysis under hypoxia

Accession	Protein name	Gene name	Log_2_ fold change	FDR
Upregulated				
Q9NUQ6	SPATS2-like protein	SPATS2L DNAPTP6 SP1224	5.3018	0.0001
P09382	Galectin-1	LGALS1	3.4357	0.0003
P14314	Glucosidase 2 subunit beta	PRKCSH G19P1	3.2139	0.0003
P61769	Beta-2-microglobulin	B2M CDABP0092 HDCMA22P	2.7460	0.0015
Q15436	Protein transport protein Sec23A	SEC23A	2.2050	0.0004
P27487	Dipeptidyl peptidase 4	DPP4 ADCP2 CD26	1.8694	0.0036
Q9UL46	Proteasome activator complex subunit 2	PSME2	1.7724	0.0272
P25786	Proteasome subunit alpha type-1	PSMA1 HC2 NU PROS30 PSC2	1.6456	0.0026
Q14103	Heterogeneous nuclear ribonucleoprotein D0	HNRNPD AUF1 HNRPD	1.6446	0.0004
P31949	Protein S100-A11	S100A11 MLN70 S100C	1.6168	0.0023
P27816	Microtubule-associated protein 4	MAP4	1.5844	0.0000
P16949	Stathmin	STMN1 C1orf215 LAP18 OP18	1.5535	0.0410
P02751	Fibronectin	FN1 FN	1.4429	0.0000
P62280	40S ribosomal protein S11	RPS11	1.3960	0.0174
Q05682	Caldesmon	CALD1 CAD CDM	1.2817	0.0125
Q08257	Quinone oxidoreductase	CRYZ	1.1956	0.0005
P37837	Transaldolase	TALDO1 TAL TALDO TALDOR	1.1582	0.0015
P62258	14-3-3 protein epsilon	YWHAE	1.1466	0.0028
P38159	RNA-binding motif protein, X chromosome	RBMX HNRPG RBMXP1	1.1085	0.0056
P42224	Signal transducer and activator of transcription 1-alpha/beta	STAT1	1.1037	0.0046
P14866	Heterogeneous nuclear ribonucleoprotein L	HNRNPL HNRPL P/OKcl.14	1.0821	0.0046
P40926	Malate dehydrogenase, mitochondrial	MDH2	1.0281	0.0050
P61254	60S ribosomal protein L26	RPL26	1.0050	0.0203
P18206	Vinculin	VCL	0.9917	0.0000
P69905	Haemoglobin subunit alpha	HBA1; HBA2	0.9644	0.0410
P39023	60S ribosomal protein L3	RPL3 OK/SW-cl.32	0.9556	0.0203
Q09666	Neuroblast differentiation-associated protein AHNAK	AHNAK PM227	0.7447	0.0003
Downregulated				
Q9Y678	Coatomer subunit gamma-1	COPG1 COPG	−8.0564	0.0013
Q9Y3D6	Mitochondrial fission 1 protein	FIS1 TTC11 CGI-135	−7.7833	0.0205
P43686	26S protease regulatory subunit 6B	PSMC4 MIP224 TBP7	−6.7742	0.0040
P40429	60S ribosomal protein L13a	RPL13A	−6.4755	0.0203
P32970	CD70 antigen (CD27 ligand)	CD70 CD27L CD27LG TNFSF7	−6.1143	0.0023
Q9Y6M1	Insulin-like growth factor 2 mRNA-binding protein 2	IGF2BP2 IMP2 VICKZ2	−5.1670	0.0104
Q02878	60S ribosomal protein L6	RPL6 TXREB1	−4.4586	0.0133
P28066	Proteasome subunit alpha type-5	PSMA5	−4.1528	0.0032
P84098	60S ribosomal protein L19	RPL19	−3.7419	0.0000
P26373	60S ribosomal protein L13	RPL13 BBC1 OK/SW-cl.46	−3.2209	0.0029
Q6PIU2	Neutral cholesterol ester hydrolase 1	NCEH1 AADACL1 KIAA1363	−2.9792	0.0000
P50914	60S ribosomal protein L14	RPL14	−2.5631	0.0480
Q14566	DNA replication licencing factor MCM6	MCM6	−2.4344	0.0203
Q13765	Nascent polypeptide-associated complex subunit alpha	NACA HSD48	−2.4075	0.0029
P55072	Transitional endoplasmic reticulum ATPase	VCP	−2.3791	0.0375
Q96C19	EF-hand domain-containing protein D2	EFHD2 SWS1	−2.3705	0.0061
O76021	Ribosomal L1 domain-containing protein 1	RSL1D1 CATX11 CSIG PBK1 L12	−2.2907	0.0339
P21980	Protein-glutamine gamma-glutamyltransferase 2	TGM2	−2.0064	0.0001
P18669	Phosphoglycerate mutase 1	PGAM1 PGAMA CDABP0006	−1.9852	0.0000
Q96KP4	Cytosolic non-specific dipeptidase	CNDP2 CN2 CPGL PEPA	−1.9098	0.0003
P07437	Tubulin beta chain	TUBB TUBB5 OK/SW-cl.56	−1.7965	0.0000
P04792	Heat shock protein beta-1	HSPB1 HSP27 HSP28	−1.7811	0.0000
O15460	Prolyl 4-hydroxylase subunit alpha-2	P4HA2 UNQ290/PRO330	−1.7067	0.0000
Q9Y265	RuvB-like 1	RUVBL1 INO80H NMP238 TIP49 TIP49A	−1.6956	0.0410
P68363	Tubulin alpha-1B chain	TUBA1B	−1.4303	0.0000
P11166	Glucose transporter 1	GLUT1 SLC2A1	−1.3864	0.0004
P36578	60S ribosomal protein L4	RPL4 RPL1	−1.3288	0.0054
P60174	Triosephosphate isomerase	TPI1 TPI	−1.2345	0.0104
P05388	60S acidic ribosomal protein P0	RPLP0	−1.2313	0.0423
P06733	Alpha-enolase	ENO1 ENO1L1 MBPB1 MPB1	−1.2069	0.0000
P05556	Integrin beta-1	ITGB1 FNRB MDF2 MSK12	−1.1859	0.0306
Q01813	6-phosphofructokinase, platelet type	PFKP PFKF	−1.0405	0.0016
P63104	14-3-3 protein zeta/delta	YWHAZ	−0.9364	0.0000
P46940	Ras GTPase-activating-like protein IQGAP1	IQGAP1 KIAA0051	−0.9120	0.0002
P07355	Annexin A2	ANXA2 ANX2 ANX2L4 CAL1H LPC2D	−0.8935	0.0000
P00338	l-lactate dehydrogenase A chain	LDHA PIG19	−0.7049	0.0004

For validating the hits obtained from LC-MS/MS and PECA analysis, we selected proteins to study with a Western blot analysis using two siRNA sequences (siPHD3#1 and siPHD3#2). In line with our LC-MS/MS results, in 786-O cells, PHD3 depletion in 786-O cells resulted in downregulation of lactate dehydrogenase A (LDHA), integrin β1, CD70 (also known as CD27L) and upregulation of malate dehydrogenase (MDH2), signal transducer and activator of transcription 1 (STAT1), fibronectin, and alpha-actinin 4 with both siRNA sequences (Fig. 1d). For integrin β1, PHD3 depletion resulted in more pronounced downregulation of the mature form (upper band) as compared to the premature form (lower band). A subset of these proteins were validated also in RCC4 cell line, including LDHA, integrin β1, MDH2, STAT1 and GLUT1. These results verified the top proteins obtained as a result of the LC-MS/MS and PECA analysis and demonstrated that the effect is not siRNA sequence specific.

### Alterations in the GO biological processes in response to PHD3 knockdown

Next, we asked if the top 91 differentially regulated proteins with FDR <0.05 and quantified from all three experiments cluster into biological functional groups. To reveal possible biological processes, the gene coding for the top proteins were analysed with several freely available gene ontology (GO) analysis tools, including the Database for Annotation, Visualization and Integrated Discovery (DAVID) and Cytoscape with the Biological Networks Gene Ontology plug-in (BINGO).

**Fig. 2:**
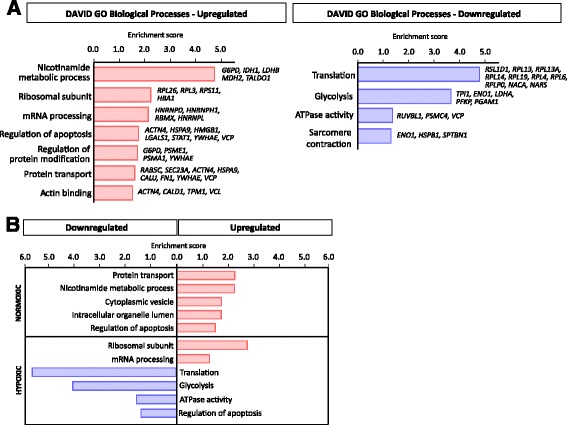
Gene ontology (GO) analysis of genes corresponding to the upregulated and downregulated proteins in response to PHD3 silencing. **a** Illustration based on the analysis performed with DAVID 6.7 from significantly changed proteins according to PECA analysis for upregulated and downregulated proteins under PHD3 knockdown. Enrichment score was used to rank the annotation groups. **b** Illustration based on the DAVID results from significantly changed proteins according to PECA analysis for normoxic and hypoxic protein groups separately. Enrichment score was used to rank the annotation groups

Functional enrichment analyses were performed for proteins downregulated or upregulated by PHD3 depletion separately, regardless of the oxygen condition. The DAVID analysis was performed with the highest classification stringency, using whole organism (*Homo sapiens*) as a background and only the functional groups with enrichment score above 1.3 were considered. The annotations for the top upregulated proteins included nicotinamide metabolic processes, ribosomal subunits, mRNA processing, regulation of apoptosis and regulation of protein modification. Likewise, protein transport and actin binding were found to be enriched in the upregulated proteins. The downregulated GO categories showed significant over-representation of translation, glycolysis, cellular ATPase activity and sarcomere contractility (Fig. 2a).

We also tested the enrichment of GO annotations separately for normoxia and hypoxia. Interestingly, under normoxia, enriched GO annotations were found only from the group of upregulated proteins (Fig. 2b). In contrast, under hypoxia, enriched GO annotations were found mostly within the group of downregulated proteins with an exception of mRNA-regulating protein groups. This could suggest that under normoxia, high levels of PHD3 suppress the expression level of given functional groups and in hypoxia, on the contrary, enhances the expression of certain functional groups. Simultaneously, we used BINGO to verify the GO annotations observed with DAVID, and the results were similar in terms of the biological processes and the direction of the regulation including the groups of nicotinamide metabolic process and translation (Additional file 2: Figure S2).

Collectively, the GO annotation results suggest that PHD3 has far more functions in ccRCC cells than are currently known and that the functions of PHD3 are linked to protein translation process, to mRNA processing and to nicotinamide cofactor (cellular glucose) metabolism.

### PHD3 regulates glucose metabolism and lactate production in ccRCC

**Fig. 3:**
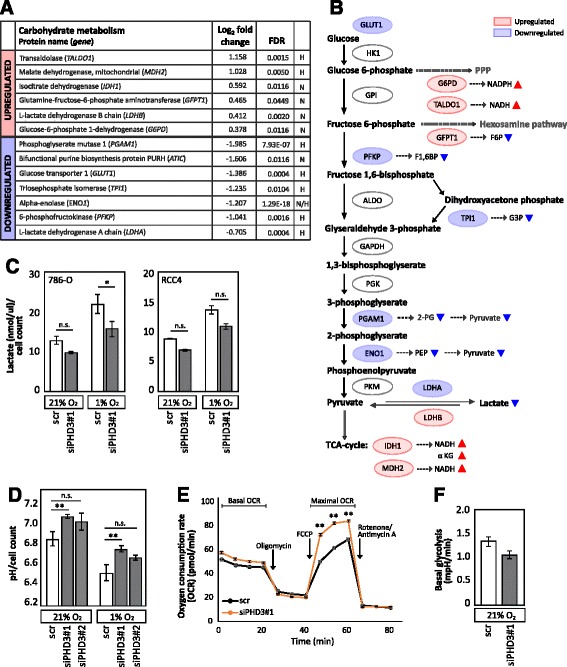
PHD3 regulates glucose metabolism of ccRCC cells. **a** PECA analysis results with log_2_ fold change and FDR. *H* hypoxic, *N* normoxic. Upregulated proteins marked by *red* and downregulated by *blue*. **b** Illustration of the glycolytic pathway. *Red ovals* represent upregulated and *blue ovals* downregulated proteins in response to PHD3 knockdown in 786-O cells according to PECA analysis. *Clear ovals* represent glycolytic enzymes not affected by PHD3 ﻿depletion based on LC-MS/MS analysis. **c** Extracellular lactate concentration normalized to cell count of 786-O and RCC4 cells in normoxia and hypoxia shows a decrease with PHD3 silencing. Quantification of three (786-O) or two (RCC4) biological replicates, mean ± SEM (*p < 0.05, *n.s.* not significant). **d** pH measured from 786-O cell culture medium in normoxia and hypoxia shows lower level of extracellular acidification with PHD3 depletion. Hydronium ion concentration was normalized to cell count and converted to pH. Quantification of three biological replicates, mean ± SEM (**p < 0.01, *n.s.* not significant). **e** Oxygen consumption rate (*OCR*) studied with Seahorse XFp Analyser shows an increase in basal OCR and in maximal OCR with PHD3 depletion. Data presented as mean ± SEM, *n* = 3 (**p < 0.01). **f** Basal glycolysis function measured with Seahorse XFp Analyser shows a decrease with PHD3 depletion. Mean ± SEM, *n* = 3. *Key*: *GLUT1* solute carrier family 2, facilitated glucose transporter member 1, *HK1* hexokinase 1, *G6PD* glucose 6-phosphate 1-dehydrogenase, *NADPH* nicotinamide adenine dinucleotide phosphate, *TALDO1* transaldolase, *GPI* glucose-6-phosphate isomerase, *PFKP* 6-phosphofructokinase, *GFPT* glutamine-fructose-6-phosphate aminotransferase, *F6P* fructose 6-phosphate, *F1,6BP* fructose 1,6-bisphosphate, *ALDO* fructose-bisphosphate aldolase, *TPI1* triosephosphate isomerase, *G3P* glyceraldehyde 3-phosphate, *GAPDH* glyceraldehyde phosphate dehydrogenase, *PGK* phosphoglycerate kinase, *PGAM1* phosphoglyserate mutase 1, *3PG* 3-phosphoglycerate, *2PG* 2-phosphoglycerate, *ENO1* Alpha-enolase, *PEP* phosphoenolpyruvate, *PKM* pyruvate kinase isoenzyme M, *LDHA* l-Lactate dehydrogenase A chain, *LDHB* l-Lactate dehydrogenase B chain, *MDH2* malate dehydrogenase, *NADH* nicotinamide adenine dinucleotide, *IDH1* isocitrate dehydrogenase, *αKG* alpha-ketoglutarate, *NOX* normoxia, *HOX* hypoxia

ccRCC has been suggested to rely on altered glucose metabolism [4, 6–8]. In line with this, a major PHD3 responsive group was found in proteins involved in glucose metabolism (GO annotations of nicotinamide cofactor metabolism and glycolysis by DAVID). A significant deregulation was seen in nine key proteins, which enable ccRCC cells to effectively utilize glycolytic pathway [4–6, 8]. Glucose-6-phosphate-dehydrogenase (G6PD), glutamine-fructose-6-phosphate aminotransferase (GFPT1), transaldolase (TALDO1), l-lactate dehydrogenase B (LDHB), isocitrate dehydrogenase (IDH1) and malate dehydrogenase (MDH2) were significantly upregulated. On the contrary, alpha-enolase (ENO1), phosphoglycerate mutase 1 (PGAM1), glucose transporter 1 (GLUT1), l-lactate dehydrogenase A (LDHA), 6-phosphofructokinase (PFKP), and triosephosphate isomerase (TPI1) and bifunctional purine synthesis protein PURH (ATIC) were found to be significantly downregulated in response to PHD3 depletion (Fig. 3a).

When investigating proteins in glycolytic pathway, a clear pattern of glycolytic pathway suppression was seen as a response to PHD3 depletion (Fig. 3b). The key glycolytic enzymes PFKP, TPI1, ENO1, PGAM1 and LDHA, as well as GLUT1, were all found to be significantly downregulated by PHD3 knockdown in hypoxic condition. PFKP is one of the major regulatory enzymes in glycolysis that function in catalyzing the irreversible conversion of fructose-6-phosphate (F6P) to fructose-1,6-bisphosphate (F1,6BP). Downregulation of PFKP is likely to result in reduced level of F1,6BP and reduced glycolytic flux. Similarly, downregulation of TPI1 and PGAM1 would result in decrease in glycolytic flux. ENO1 catalyzes the conversion of 2-phosphoglycerate (2-PG) into phosphoenolpyruvate (PEP) and thus the downregulation by PHD3 silencing leads to decreased levels of PEP and pyruvate. It is notable that ENO1 is the most significantly downregulated protein both in normoxic and hypoxic condition as a response to PHD3 depletion (log_2_ fold change 1.21; FDR 1.29^−18^). In addition to glycolytic enzymes, GFPT1 that links glutamine metabolism to glycolytic pathway was found upregulated in response to PHD3 depletion. Thus, upregulated GFPT1 suggests increased flux of glucose to the hexosamine pathway while suppressing the levels of glycolytic F6P.

Noticeably, lactate dehydrogenases LDHA and LDHB were found to react in opposite directions in response to PHD3 depletion; LDHA being downregulated under hypoxia while LDHB being upregulated under normoxia. LDH enzymes convert pyruvate into lactate in a bi-directional manner—LDHA from pyruvate to lactate and LDHB from lactate to pyruvate (reviewed in [33]). Consequently, both downregulated LDHA and upregulated LDHB are likely to result in decreased pyruvate to lactate conversion. In contrast to glycolytic enzymes, TCA cycle enzymes IDH1 and MDH2 were found upregulated as a response to PHD3 depletion.

Motivated by the finding of suppressed glycolytic enzymes in response to PHD3 silencing, we measured lactate concentration and pH from the cell culture medium of normoxic and hypoxic cells. In line with the downregulated LDHA, extracellular lactate concentration was markedly reduced with PHD3 depletion in 786-O cells and even more pronounced effect was detected under hypoxic condition (normalized to cell count). Similar results were seen also with RCC4 cells (Fig. 3c). In agreement with this, the pH measured from the media was found significantly higher in PHD3-depleted cells by using two individual siRNA sequences targeting PHD3, suggesting a lower level of acidification in the cell culture medium (Fig. 3d). Furthermore, we investigated the oxygen consumption rate (OCR) and glycolysis function on 786-O by using Seahorse XFp Analyser (Agilent Technologies). PHD3 silencing increased both basal OCR and maximal OCR measured after stimulating the cells with mitochondrial membrane uncoupling agent FCCP (carbonyl cyanide-4 trifluoromethoxy phenylhydrazone) that allows maximal electron flow through the electron transport chain and thus the maximal oxygen consumption at the mitochondria (Fig. 3e). In addition, basal glycolysis measured by Seahorse Analyser was decreased with PHD3 silencing (Fig. 3f). These data further functionally validate the results from discovery proteomics as well as extracellular lactate and pH measurements. In summary, the downregulation of the key glycolytic enzymes in response to PHD3 depletion results in suppression of glycolysis and lactate production and in a metabolic shift towards oxidative phosphorylation. This is most prominent under hypoxia.

### PHD3 regulates ribosomal subunits and protein translation in ccRCC

**Fig. 4:**
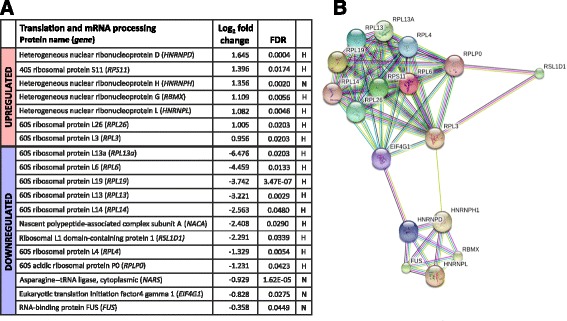
Functional groups of translation and mRNA processing are regulated in response to PHD3 silencing. **a** PECA analysis results of LC-MS/MS data with log_2_ fold change and FDR. *H* hypoxic, *N* normoxic. Upregulated proteins marked by *red* and downregulated by *blue*. **b** String analysis showing known interactions between the deregulated proteins by PHD3 knockdown from the group of translation and mRNA processing

We further scrutinized the cellular processes that showed major regulation in response to PHD3 depletion based on the GO categorizing. One group that showed major alteration were proteins involved in translation. Within the group, a number of ribosomal protein subunits were significantly downregulated as response to PHD3 knockdown (Fig. 4a). The most downregulated ribosomal proteins based on their log_2_ fold change were RPL13A, RPL6, RPL19 and RPL13, which all are components of 60S large subunit. Fewer RPLs were upregulated, including RPL3 and RPL26, as well as RPS11, a 40S small subunit component. Besides ribosomal proteins, asparagine—tRNA ligase (NARS), RNA-binding protein FUS, eukaryotic translation initiation factor 4 gamma 1 (eIF4G1) and nascent polypeptide-associated complex subunit (NACA) were found to be significantly downregulated. As all above proteins are involved in different steps of the translation and protein maturing processes, the data implies that PHD3 is involved in maintenance processes of the ribosomal integrity and protein translation in ccRCC cells.

Supporting the effect of PHD3 in protein production, another group that showed intriguing deregulation in response to PHD3 knockdown was the mRNA processing. Significant upregulation in the group of post-transcriptional regulators and the heterogeneous nuclear ribonucleoproteins (hnRNPs) D, H, L and G was detected. These proteins function mainly in the regulation of mRNA metabolism and at different stages of mRNA maturing process. The most upregulated protein was found to be hnRNPD, also known as RNA-binding protein AUF1. As demonstrated by STRING analysis, PHD3 maintains a lower level of a large group of ribonuclear proteins that interact with each other (Fig. 4b). The data suggests that PHD3 controls a large group of mRNA maturation and protein translation regulating enzymes, in particularly under hypoxia.

**Fig. 5:**
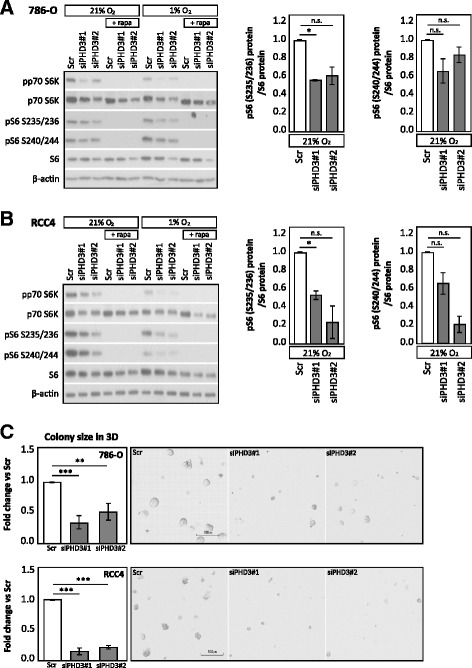
PHD3 depletion leads to impaired ccRCC cell growth and decreases the phosphorylation of p70 S6K and S6 ribosomal proteins. **a** Western blot analysis of 786-O cells with two siRNA sequences targeting PHD3 under normoxia and hypoxia shows decrease in phosphorylation of p70 S6K and S6 ribosomal protein. *Right-hand* graphs show phosphorylated (S235/236 and S240/244) pS6 ribosomal protein normalized to total S6 protein. Quantification of two biological replicates, mean ± SEM, fold change to Scr (*p < 0.05, *n.s.* not significant). **b** Western blot analysis of RCC4 cells with PHD3 depletion under normoxia and hypoxia decreases the phosphorylation of p70 S6K and S6 ribosomal protein. Quantification of two biological replicates, mean ± SEM, fold change to Scr (*p < 0.05, *n.s.* not significant). **c** 786-O and RCC4 3D colony forming in Matrigel®. Quantification of three (786-O) or two (RCC4) biological replicates with triplicate wells for each siRNA treatment, mean ± SEM, fold change to Scr (**p < 0.01, ***p < 0.001). Representative images in the panel acquired by using ×4 objective, *scale bar* 100 μm

Components of translational machinery, including all ribosomal proteins and translation initiation and elongation factors, belong to the group of TOP (5′ Terminal Oligo Pyrimidine motif) mRNAs. The translation of TOP mRNAs is strictly regulated by amino acid and nutrient availability. Previous studies have shown that TOP mRNAs are directly regulated by the phosphorylation of p70 S6 kinase (p70 S6K) and its target S6 ribosomal protein (reviewed in [34]). Next, we asked if the phosphorylation of p70 S6K and the downstream effector S6 ribosomal protein is affected by PHD3 depletion, as we saw the protein level of numerous translational machinery components downregulated in LC-MS/MS results. Interestingly, phosphorylation of p70 S6K (T389) was downregulated with PHD3 silencing in both 786-O and RCC4 cells (Fig. 5a, b). However, also, total p70 S6K protein level was decreased as a result of PHD3 depletion, suggesting that both the expression level and activation of p70 S6K are affected by PHD3. In line, also, phosphorylation of S6 ribosomal protein (pS6 S235/236 and S240/244) was similarly downregulated by PHD3 depletion while total S6 ribosomal protein remained unaffected. Rapamycin treatment was used as a control for inhibition of mTOR signalling. Importantly, T389 phosphorylation of p70 S6K has been shown to be a direct target of mTORC1 [35], which could suggest an involvement of PHD3 in mTORC1 activity and downstream signalling. In conjunction, the findings suggest a possible mechanism for PHD3 in the regulation of the protein-level expression of the translational machinery components.

Finally, we asked whether the PHD3-mediated regulation of translational machinery and cellular energy metabolism has an effect on ccRCC cell growth. In our previous studies, we have shown that PHD3 depletion causes cell cycle arrest at G1 in several carcinoma cell lines, including 786-O [18, 19]. To verify the cell cycle arrest in ccRCC, we determined the effect of PHD3 depletion on cell cycle of another ccRCC cell line (RCC4). In line with the previously published results on 786-O cell line, the FACS analysis showed that depleting PHD3 in RCC4 cell line causes G1 arrest both in normal oxygen pressure (21% O_2_) and in hypoxia (1% O_2_) (Additional file 3: Figure S3A), Additional file 4.

We have previously shown that PHD3 depletion reduced proliferation in squamous cell carcinoma (SCC) cells measured by BrdU incorporation [18]. To further study the effects of the cell cycle arrest in ccRCC cells, we followed the proliferation of cells treated with two distinct PHD3-targeting siRNAs for 96 h. Indeed, both PHD3-targeting siRNAs reduced the proliferation of 786-O. The reduction was even more prominent in RCC4 cells (Additional file 3: Figure S3B). However, the viability of the cells was not affected (data not shown) nor was the proportion of sub-G1 cells (Additional file 3: Figure S3C). However, as we investigated the 3D colony growth of 786-O and RCC4 cells by using Matrigel® matrix, significant reduction in colony size with PHD3 silencing was seen in both cell lines (Fig. 5c). This verified the essential role of PHD3 in ccRCC cell growth that is in line with the effects of PHD knockdown on protein expression in metabolic, translation and mTOR downstream pathways.

## Discussion

The oxygen sensing prolyl hydroxylase family member PHD3 displays high expression levels in ccRCC tumours and derived cell lines [23, 24]. Recent studies have suggested a number of both hydroxylase-dependent and hydroxylase-independent roles for PHD3 also beyond the HIF pathway. In cancer cells, these involve the regulation of cellular survival mechanisms [15–19] and the regulation of metabolism by individual genes [20–22]. However, the more global function of elevated PHD3 expression in ccRCC has remained enigmatic.

Here, we studied the role of PHD3 in 786-O ccRCC cells using discovery proteomics approach and peptide-level averaging quantification method, PECA [27]. The 786-O cells have inactivated pVHL and truncated HIF-1α. Thus, on the HIF pathway, 786-O cells retain only HIF-2α, the best characterized PHD3 target. By using LC-MS/MS, approximately hundred proteins were found to be significantly deregulated in response to PHD3 silencing in all of the three biological replicates. For PHD3 silencing, we used a sequence well characterized by us and others [18, 19, 30–32]. Importantly, the results were further validated with the second independent siRNA sequence by using expression analysis of selected proteins, some of which belong to the highlighted glucose metabolism pathway. A subset of the selected proteins were also validated in another *VHL*-mutated ccRCC cell line, RCC4 expressing both HIF-1α and HIF-2α.

Our data indicates that PHD3 affects major cellular biological processes involved in glucose metabolism, mRNA processing and protein translation. Other functional groups annotated included apoptosis, protein modification process, protein transport, actin cytoskeleton and ATPase activity. Moreover, under hypoxia, the response to PHD3 knockdown was more widespread than under normoxic condition illustrated by both the number of regulated proteins and by the range of the quantified protein expression. This could suggest that despite the high expression level of PHD3 in both oxygen conditions, depletion of PHD3 has more pronounced effects under restricted oxygen availability. While a large amount of proteins within the annotated functional groups were detected, a targeted MS approach would be required for detecting specific very low abundance proteins among these groups.

ccRCC cells are laden with glycogen and lipid which implies altered fatty acid and glucose metabolism [36]. Moreover, ccRCC tumours and derived cells lines have been shown to consistently display increased expression of the glycolytic pathway enzymes [4, 6, 37]. Interestingly, major proteome-level changes affected by PHD3 depletion were found in glucose metabolic pathway. Our findings imply that PHD3 silencing leads to suppression of glycolytic pathway as well as decreased conversion of pyruvate to lactate demonstrated by the regulation of nine glycolytic enzymes. Key glycolytic enzymes including PFKP, TPI1, ENO1, PGAM1 and LDHA together with glucose transporter GLUT1 were all found to be significantly downregulated. Interestingly, PGAM1 that has been shown upregulated in ccRCC tumours was found to downregulated in response to PHD3 depletion [38].

Tumour acidification and lactate levels have been shown to correlate with increased metastasis and poor patient outcome by promoting tumour inflammation and activating angiogenesis [33, 39]. Noticeably, the proteome-level data suggests decreased pyruvate to lactate conversion in response to PHD3 knockdown illustrated by LDHA downregulation under hypoxia and LDHB upregulation under normoxia. LDHA converts pyruvate into lactate and LDHB lactate to pyruvate, thus having key function in the regulation of microenvironmental pH. LDHA has shown to be overexpressed in many cancers and to play a crucial role in tumour proliferation, invasion and metastasis whereas the role of LDHB remains more elusive (reviewed in [33]). In line with the proteome-level data, we found decreased extracellular lactate levels in response to PHD3 silencing in both cell lines and as previously shown by others in RCC4 [21]. pH rise with two individual siPHD3 sequences further supports the decreased lactate production. Whereas Luo et al. suggested the metabolic effects of PHD3 would be mediated through HIF-1α, in our setting using 786-O cells, HIF-1α is truncated and thus unlikely downstream mediator of PHD3 depletion. It is possible however that HIF-2α could mediate the metabolic shift seen in PHD3-depleted ccRCC cells. PHD3 has also been shown to regulate hepatic glucose metabolism through regulating HIF‐2α [40]. Moreover, since several non-HIF targets have been reported for PHD3, it is feasible that the regulation of the proteins found based on the discovery proteomics approach are not directly regulated by PHD3, but via an upstream effector responsible for overall regulation of cellular energy metabolism.

In contrast to decreased levels of glycolytic enzymes, we found TCA cycle enzymes IDH1 and MDH2 elevated in response to PHD3 depletion. As these enzymes are essential in cellular NADH coenzyme production, the increase would likely result in elevated levels of NADH and α-ketoglutarate. Interestingly, α-ketoglutarate is considered as a rate-limiting factor in TCA cycle and also an essential co-substrate of PHD enzymes [41]. This suggests an enhancement of TCA cycle by PHD3 knockdown while decreasing the flux towards lactate. The oxidative metabolism was further studied by using Seahorse XFp Analyser measuring oxygen consumption rate (OCR) in 786-O cells. Indeed, in line with the previously reported data for RCC4 cells [21], siPHD3 treatment enhanced basal and maximal OCR. Our data further validate the previous findings by others. Moreover, it suggests an HIF-1α-independent role for PHD3 in the regulation of glycolysis and lactate production as it occurs in HIF-1α deficient cell line.

Interestingly, another major functional group responding to PHD3 silencing was protein translation and ribosomal subunits. Among these proteins were ten ribosomal proteins that together with ribosomal RNA (rRNA) and transfer RNA (tRNA) constitute the ribosomal subunits and catalyze the mRNA-directed protein translation. In addition to ribosomal proteins, eIF4G1, FUS, NARS and NACA that are involved in different steps of the translation and protein maturing processes were found downregulated by PHD3 silencing. Given such large number of proteins regulated by PHD3 depletion, it is likely that PHD3 has a crucial function in maintaining the integrity of translational machinery particularly under hypoxia in ccRCC. In line, the overexpression of certain ribosomal proteins has previously been linked to several types of cancer [42].

Closely related to protein translation, hnRNPs D, H, L and G were found to be significantly upregulated by PHD3 silencing. The function of hnRNPs lies in mRNA processing that includes events such as pre-mRNA alternative splicing, tissue-specific regulation of gene transcription and regulation of mRNA stability. hnRNPD, also known as RNA-binding protein AUF1, is known to regulate the mRNA stability of many proteins involved in inflammatory response and developmental processes, as well as oncogenes [43]. The importance of PHD3 in cellular protein production is strongly suggested as both hnRNPs and RPLs are essential in the regulation of protein expression. In line with RPL regulation, PHD3 knockdown mainly targeted the hnRNPs under hypoxia suggesting a specific role for PHD3 under restricted oxygen availability. In keeping with this, PHD3 has been demonstrated to retain its activity under moderate (1%) hypoxia, e.g. towards HIF-2α (reviewed in [14]).

Further studies on the upstream regulators of the translation machinery components revealed that phosphorylation of both p70 S6K and its downstream target S6 ribosomal protein was reduced by PHD3 silencing. Phosphorylation of p70 S6K on T389 by mTORC1 and subsequent phosphorylation of S6 ribosomal protein have been shown to be responsible for the regulation of the TOP mRNA translation, a strictly regulated group of translational machinery components including all ribosomal proteins (reviewed in [34]). However, also, total p70 S6K protein expression was slightly decreased with PHD3 silencing, which could indicate a direct effect of PHD3 on p70 S6K, but further studies are needed to determine the detailed mechanism. p70 S6K and phosphorylation of S6 ribosomal protein are also crucial downstream effectors of mTOR in regulation of cell growth and cell cycle (reviewed in [34]). A reduction in phosphorylation of both p70 S6K and S6 ribosomal protein is in line with our findings on the cell cycle arrest in response to PHD3 depletion [19]. Inhibition of mTOR signalling by small molecular inhibitor has been shown to reduce ccRCC cell clonogenicity and proliferation, but not affecting cell viability or apoptosis [44]. In line, we saw a similar effect on cell proliferation and colony formation in 3D by PHD3 depletion but no effect on cell viability or apoptosis. Finally, the suppression of translational machinery by the reduced availability of the amino acids causes cell cycle block at G1/S transition [45]. As we have previously shown that PHD3 depletion impairs G1/S transition in ccRCC, one could speculate that the mechanism of cell cycle arrest could involve the suppression of mTOR downstream signalling. As PHD3 depletion reduces the activation of the key mTORC1 downstream effectors and has similar effects on cell growth than mTOR inhibition, it is plausible that PHD3 has a role in mTOR signalling pathway regulation.

One cannot rule out that both the suppression of glycolytic pathway and the suppression of translation machinery in response to PHD3 silencing are linked together via the same upstream effector. mTOR signalling is a major pathway regulating cellular growth and also metabolism. In case of PHD3 regulating the downstream effectors of mTOR, it is possible that also the effect on glucose metabolism could be mediated by the regulation of mTOR signalling in ccRCC. Importantly, TOP mRNAs are translationally repressed when cells are subjected to stress by their environment such as deprivation of energy, nutrients and oxygen deprivation (reviewed in [46]. To conclude, high PHD3 expression in ccRCC cells results in enhanced translation of the ribosomal proteins and other components of the protein synthesis machinery, thus protecting cancer cells from the cell cycle arrest caused by the environmental stress and enabling cells to overcome constrains of the hostile environment. In addition, high PHD3 expression would lead to enhanced glycolytic activity and increased lactate production, thus contributing to tumour progression. Further studies are needed to determine the detailed mechanism behind the regulation of the translational machinery and glucose metabolism by PHD3 and to investigate the effects across different cancer types.

## Conclusions

Here, we report that the PHD3 oxygen sensor regulates glucose metabolism as well as translation at multiple levels in ccRCC cells as studied with discovery proteomics and label-free quantification of proteome data. Our results imply the involvement of PHD3 in the maintenance of high glycolytic rate and lactate production in ccRCC by regulating the expression of most key glycolytic enzymes. Moreover, PHD3 is required to maintain the expression of translational machinery components and to regulate post-transcriptional processes via mRNA processing factors. Our findings highlight the multi-functionality of PHD3 in ccRCC biology beyond its HIF prolyl hydroxylase activity. Interestingly, under reduced oxygen availability, PHD3 knockdown illustrates more prominent and a greater number of protein identifications as compared to normoxia. Further studies are required to understand whether PHD3 functions by regulating the expression of glycolytic enzymes and translational machinery via a common upstream effector.

## Additional files

Additional file 1: Figure S1. Box plots visualizing peptide log fold changes of upregulated (red) and downregulated (blue) proteins between siPHD3 and Scr samples both in (A) normoxic and (B) hypoxic conditions. The box contains fold changes of 50% of the peptides belonging to each protein and the whiskers extend to minimum and maximum observed fold change. Uniprot accession number is used as a protein identification. (PDF 46 kb)

Additional file 2: Figure S2. BINGO GO annotation analysis of genes corresponding to the upregulated and downregulated proteins in response to PHD3 silencing. Annotation groups scored by the corrected *p* value for over-representation. (PDF 51 kb)

Additional file 3: Figure S3. The effect of PHD3 depletion on ccRCC cell cycle, proliferation and apoptosis. (A) Cell cycle analysis of 786-O and RCC4 cells with PHD3 depletion under normoxia and hypoxia showing G1 arrest. Quantification of four (786-O) or six (RCC4) biological replicates, mean ± SEM (*p < 0.05, **p < 0.01). (B) Incucyte® Live Cell Analysis of ccRCC cells treated with two distinct siRNA sequences targeting PHD3 shows reduced proliferation of 786-O and RCC4 cells in response to PHD3 depletion. Representative growth curves of three individual experiments, mean values of four wells ± SEM (*p < 0.05, **p < 0.01, marked above the curve for siPHD3#1 and below the curve for siPHD3#2). (C) FACS analysis of cells in sub-G1 phase representing the apoptotic portion of the cell population. Quantification of seven (786-O) or six (RCC4) biological replicates, mean ± SEM (*p < 0.05, n.s. not significant). (PDF 128 kb)

Additional file 4: Supplementary methods. (DOCX 12 kb)

## Abbreviations

ccRCC: Clear cell renal cell carcinoma
FDR: False discovery rate
GO: Gene ontology
HIF: Hypoxia-inducible factor
LC-MS/MS: Liquid chromatography tandem-mass spectrometry
mTOR: Mechanistic target of rapamycin
PHD: Prolyl-4-hydroxylase domain protein
pVHL: von Hippel-Lindau protein
