# Supplementary figures and images for: HIF prolyl hydroxylase PHD3 regulates translational machinery and glucose metabolism in clear cell renal cell carcinoma
Source: Cancer Metab. 2017 Jul 4;5:5. doi: 10.1186/s40170-017-0167-y (PMC5496173; doi:10.1186/s40170-017-0167-y)

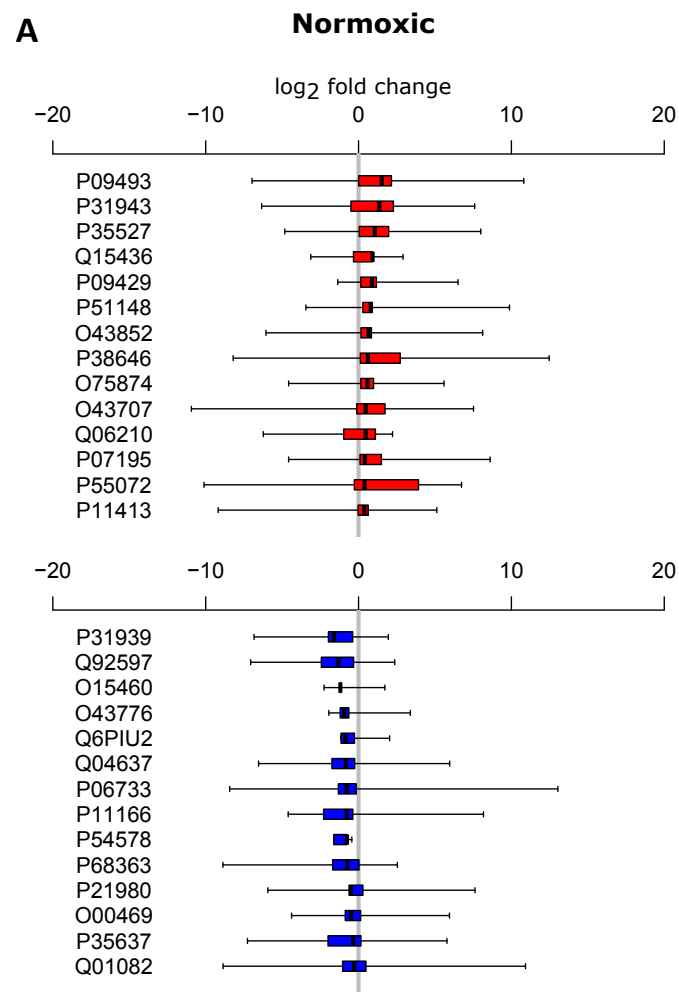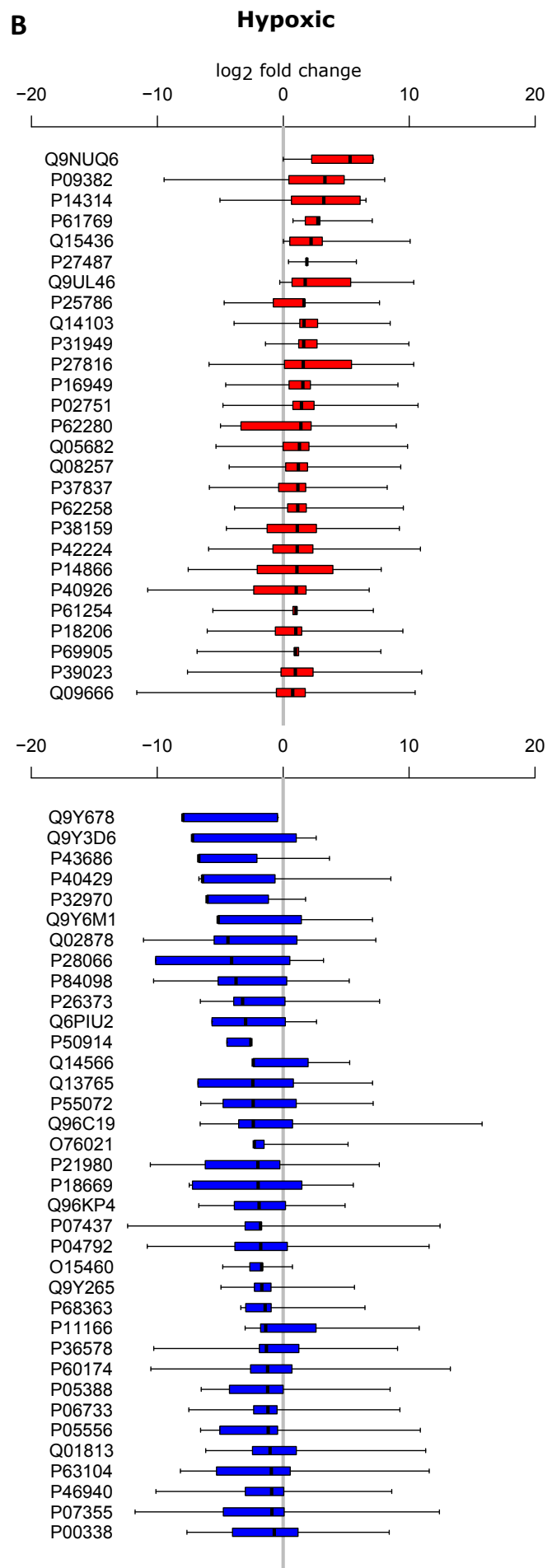

Supplement: Supplementary file 1 — Box plots visualizing peptide log fold changes of upregulated (red) and downregulated (blue) proteins between siPHD3 and Scr samples both in (A) normoxic and (B) hypoxic conditions. The box contains fold changes of 50% of the peptides belonging to each protein and the whiskers extend to minimum and maximum observed fold change. Uniprot accession number is used as a protein identification. (PDF 46 kb) [file 40170_2017_167_MOESM1_ESM.pdf]

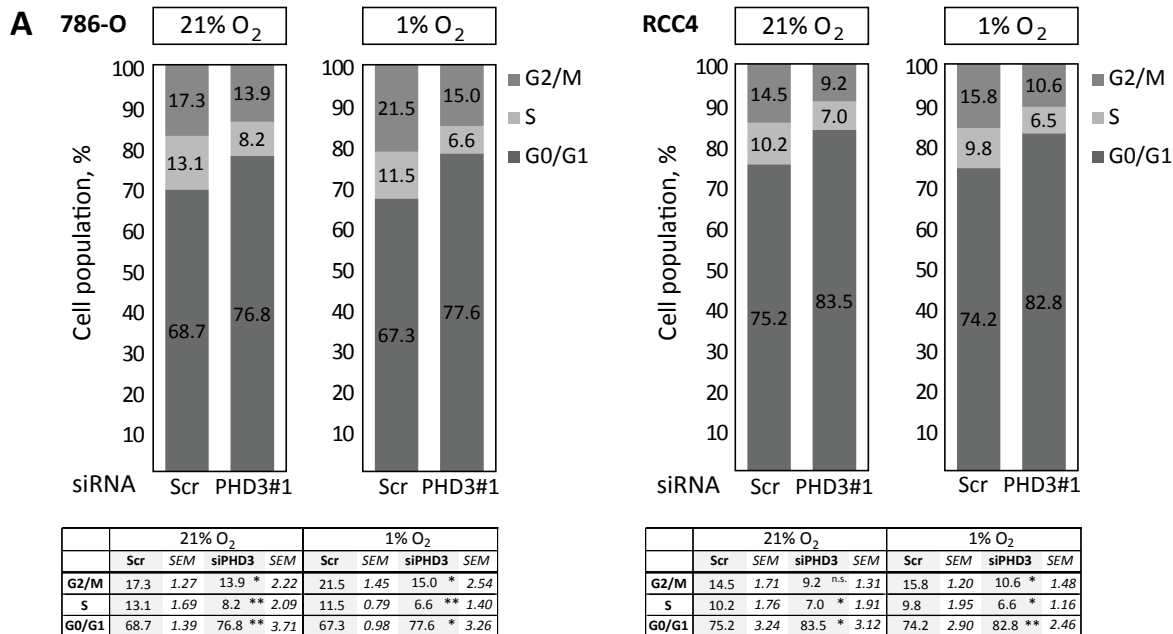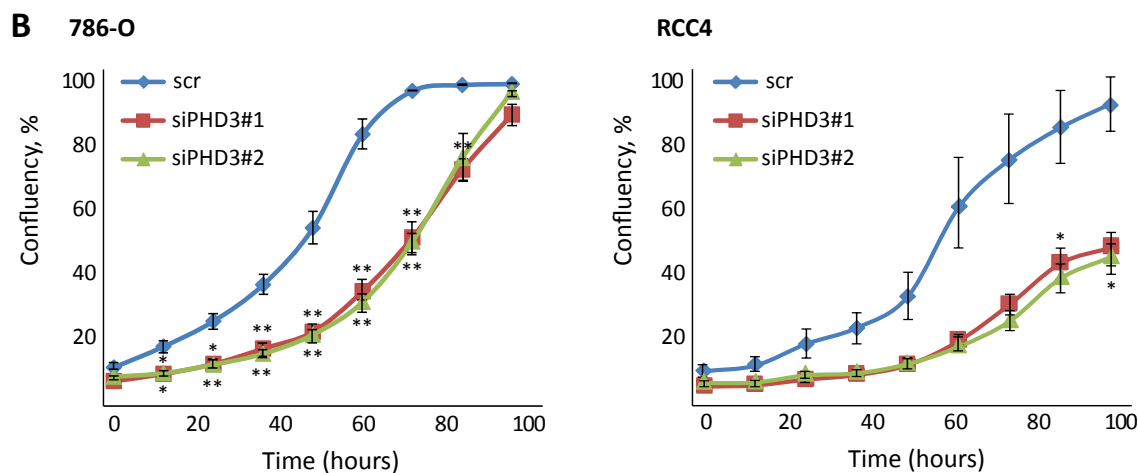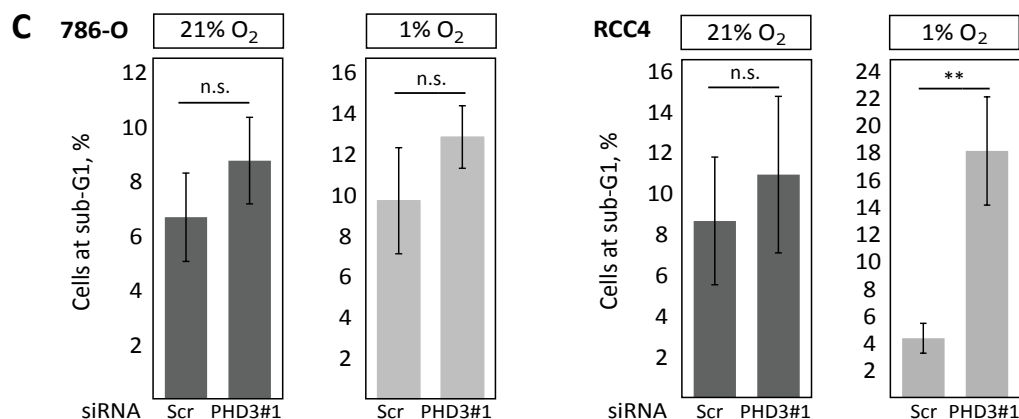

Supplement: Supplementary file 3 — The effect of PHD3 depletion on ccRCC cell cycle, proliferation and apoptosis. (A) Cell cycle analysis of 786-O and RCC4 cells with PHD3 depletion under normoxia and hypoxia showing G1 arrest. Quantification of four (786-O) or six (RCC4) biological replicates, mean ± SEM (*p < 0.05, **p < 0.01). (B) Incucyte® Live Cell Analysis of ccRCC cells treated with two distinct siRNA sequences targeting PHD3 shows reduced proliferation of 786-O and RCC4 cells in response to PHD3 depletion. Representative growth curves of three individual experiments, mean values of four wells ± SEM (*p < 0.05, **p < 0.01, marked above the curve for siPHD3#1 and below the curve for siPHD3#2). (C) FACS analysis of cells in sub-G1 phase representing the apoptotic portion of the cell population. Quantification of seven (786-O) or six (RCC4) biological replicates, mean ± SEM (*p < 0.05, n.s. not significant). (PDF 128 kb) [file 40170_2017_167_MOESM3_ESM.pdf]
