# Supplementary material for: HIF prolyl hydroxylase PHD3 regulates translational machinery and glucose metabolism in clear cell renal cell carcinoma
Source: Cancer Metab. 2017 Jul 4;5:5. doi: 10.1186/s40170-017-0167-y (PMC5496173; doi:10.1186/s40170-017-0167-y)
Supplement: Supplementary file 2 — BINGO GO annotation analysis of genes corresponding to the upregulated and downregulated proteins in response to PHD3 silencing. Annotation groups scored by the corrected p value for over-representation. (PDF 51 kb) [file 40170_2017_167_MOESM2_ESM.pdf]

BINGO GO Biological Process - Upregulated	Corr p-value
**Nicotinamide metabolic process**<br>*TALDO1, IDH1, MDH2, G6PD*	1.59E-06
**Regulation of cellular component organization**<br>*HMGB1, DPP4, ACTN4, LGALS1, MAP4, TPM1, RAB5C, FN1*	3.64E-05
**Cellular response to chemical stimulus**<br>*HMGB1, STAT1, LGALS1, TPM1, G6PD, VCP, CRYZ*	4.26E-05
**Protein transport**<br>*SEC23A, ACTN4, RAB5C, VCP, HSPA9, HMGB1, DPP4, VCL, TPM1, YWHAE, FN1, CALD1*	1.26E-04
**Regulation of apoptosis**<br>*HMGB1, TPM1, VCP*	6.03E-04

BINGO GO Biological Process - Downregulated	Corr p-value
**Translation**<br>*RPL6, RPL19, RPL14, RPL13, RPL13A, RPL4, RPLP0, RSL1D1, NACA, NARS*	5.14E-09
**Protein metabolic process**<br>*USP14, NACA, PLOD2, P4HA2, RUVBL1, PSMA5, RPL6, RPL19, RPL14, RPL13, RPL13A, RPL4, RPLP0, RSL1D1, NCEH1, TGM2, VCP, PSMC2, CNDP2, MCM6, NACA, NARS, SPTBN1*	2.14E-07
**Glycolysis**<br>*ENO1, TPI1, PFKP, PGAM1*	2,16E-04
