# Supplementary material for: HIF prolyl hydroxylase PHD3 regulates translational machinery and glucose metabolism in clear cell renal cell carcinoma
Source: Cancer Metab. 2017 Jul 4;5:5. doi: 10.1186/s40170-017-0167-y (PMC5496173; doi:10.1186/s40170-017-0167-y)
Supplement: Supplementary file 4 — Supplementary methods. (DOCX 12 kb) [file 40170_2017_167_MOESM4_ESM.docx]

**SUPPLEMENTARY METHODS**

**Pathway analysis**

Cytoscape (version 3.3.0) with the Biological Networks GO plug-in (BINGO, version 3.0.3) [29] was used to identify enriched GO biological processes. The analysed protein coding genes were compared against a reference GO annotations (Homo sapiens), with p-value <0.05 and FDR correction (Benjamini and Hochberg). Hypergeometric test was used for over-representation.

**FACS analysis**

For cell cycle analysis siRNA treated cells were incubated 24 hours to reach 50-60 % confluence, treated for another 24 hours in normoxic (21% O_2)_ or in hypoxic (1% O_2_) condition, fixed with 70% ethanol and stained with propidium iodide. Cell cycle analysis was performed using flow cytometer (BD FACSCalibur, BD Biosciences) and BD CellQuest™ Pro software.

The proportion of the cells considered as apoptotic was measured determining the fraction of cells in sub-G1 from the flow cytometry data.

**Cell proliferation**

To follow cell proliferation siRNA treated cells were plated on 96-well plates, four wells for each siRNA. After 24 hours the well plates were placed into Incucyte® Live-Cell Analysis System (Essen BioScience) for 96 hours. The wells were scanned every 12 hours, and the proliferation rate was determined as confluency accordingly.
